# Troponin T Elevation After Percutaneous Left Atrial Appendage Occlusion

**DOI:** 10.3389/fcvm.2021.721224

**Published:** 2021-10-01

**Authors:** Xiaoyan Wang, Xueying Chen, Yong Ye, Juan Peng, Jinyi Lin, Xin Deng, Li Lin, Jieyun You, Xingxu Wang, Daxin Zhou, Qingxing Chen, Junbo Ge

**Affiliations:** ^1^Shanghai Institute of Cardiovascular Diseases, Zhongshan Hospital, Institute of Biomedical Science, Fudan University, Shanghai, China; ^2^Department of Cardiovascular Medicine, East Hospital, Tongji University School of Medicine, Shanghai, China

**Keywords:** cardiac troponin T, left atrial appendage occlusion, adverse events, Watchman® -device, LAmbre device

## Abstract

**Background:** Cardiac troponin T (cTNT) has been widely used in detecting cardiac damage. Elevated cTNT level has been reported to be associated with increased mortality in multiple cardiac conditions. It is not uncommon to observe an increased level of cTNT in patients after left atrial appendage occlusion (LAAO). The objective of the study is to study the incidence, significance, and factors associated with cTNT elevation after LAAO.

**Methods:** We prospectively included patients who underwent LAAO from January 2019 to July 2020 in Fudan Zhongshan Hospital. Patients were divided into those with elevated cTNT after procedure and those with normal postprocedure cTNT. All individuals were followed up for 1 year. The primary outcome is major adverse cardiovascular events, which include myocardial infarction, heart failure, cardiac death, and stroke. The second outcome is periprocedure complication, including chest pain, tachycardia, cardiac tamponade, change of electrocardiograph, and atrial thrombus.

**Results:** A total of 190 patients were enrolled. Of the patients, 85.3% had elevated cTNT after LAAO, while 14.7% of them did not. Exposure time, dosage of contrast, types of devices, shapes, and sizes of LAA could contribute to elevated postprocedure cTNT. We found that patients with a Watchman device were more likely to have elevated postprocedure cTNT than those with a Lambre device (89.2 vs. 76.7%, *p* = 0.029). LAAO shapes were associated with cTNT levels in patients with a Watchman device, while the diameter of the outer disc and LAA depth mattered for the Lambre device. There was no significant difference in the primary and second outcome between the two groups (*p*-value: 0.619, 0.674).

**Conclusion:** LAAO was found to be commonly accompanied with cTNT elevation, which might not to be related to the complications and adverse cardiac outcomes within 1 year of follow-up. Moreover, eGFR at baseline, exposure time, dosage of contrast, types of LAAO device, and LAA morphology could contribute to cTNT elevation.

## Introduction

Cardiac troponin T (cTNTs) are part of the cardiac contractile mechanism of the cardiac muscle, and they are highly sensitive in detecting minimal myocardial injury (1). Higher cTNT levels can be found in various conditions, such as percutaneous coronary intervention (PCI) (2, 3), coronary artery bypass grafting (CABG) (1, 4, 5), radiofrequency catheter ablation (RFA) (6–8), automatic implantable cardioverter defibrillator (ICD) (9, 10), pacemaker lead insertion (11), and have been shown to be associated with worse clinical outcomes (1, 12, 13).

However, different procedures affect levels of cTNT differently. For cardiac procedures like PCI and CABG, elevated cTNT levels after procedure is associated with worse clinical outcomes (2–5); however, for other cardiac procedures, increased postprocedure cTNT levels do not necessarily affect adverse clinical outcomes (8). It remains controversial whether increased cTNT values after cardiac procedure affect long-term adverse events.

Left atrial appendage occlusion (LAAO) has emerged as a new strategy to prevent stroke in patients with atrial fibrillation (AF) (14, 15). It is applied in AF patients who have relative contraindication to oral anticoagulants (16). For LAAO, little remains unknown about the changes of cTNT and its effect on clinical outcomes after LAAO procedure.

The objective of the study is to study the incidence, significance, and factors associated with cTNT elevation after LAAO.

## Methods

Our study is a single-center, prospective study. Postoperative cTNT refers to cTNT 12–24 h after LAAO.

### Study Population

Patients who underwent LAAO from January 2019 to July 2020 in Fudan Zhongshan Hospital were consecutively selected. Based on the cTNT levels, eligible patients were separated into elevated cTNT group and normal cTNT group. We further divided patients into two groups based on the LAAO devices: Watchman (Boston Scientific, Plymouth, MN, USA) and Lambre [Lifetech Scientific (Shenzhen) Co. Ltd., Shenzhen, China]. Within each group, we compared the LAA sizes and shapes.

Baseline characteristics of the patients included age, male, hypertension, diabetes, hyperlipidemia, history of cerebrovascular disease, coronary heart disease, structural heart disease, heart failure, history of radiofrequency ablation, history of malignant tumor, preprocedure cTNT, hemoglobin (HB), white blood cell (WBC), endogenous glomerular filtration rate (eGFR), and left ventricular ejection fraction (LVEF). The intraoperative information included types of left atrial appendage, morphological characteristics of the left atrium, and diameter and depth of the left atrial appendage.

The study was approved by the Institutional Review Board of Zhongshan Hospital, Fudan University, Shanghai, China.

### Clinical Outcomes and Definitions

The primary endpoint of this study is 1-year major adverse cardiovascular events, including myocardial infarction, heart failure, cardiac death, and stroke. The secondary endpoint is the perioperative adverse events, including chest pain, tachycardia, cardiac tamponade, change in electrocardiograph, and atrial thrombus. According to our hospital lab protocol, elevated cTNT is defined as the level of cTNT ≥0.03 μg/L, and normal cTNT is defined as cTNT <0.03 μg/L. Mild-elevated cTNT is defined as post-cTNT higher than 0.03 but lower than 0.09 μg/L. Severe-elevated post-cTNT is defined as ≥0.09 μ g/L. Myocardial infarction is defined by an elevation of cTn values >5 times of the 99th percentile URL in patients with normal baseline values. Patients with elevated preprocedural cTn values, in whom the preprocedural cTn level is stable (≤ 20% variation) or falling, must meet the criteria for a >5- or >10-fold increase and manifest a change from the baseline value of >20%. In addition, patients have at least one of the following: (1) new ischemic ECG changes (this criterion is related to type 4a MI only), (2) development of new pathological Q waves, (3) imaging evidence of loss of viable myocardium that is presumed to be new and in a pattern consistent with an ischemic etiology, and (4) angiographic findings consistent with a procedural flow-limiting complication such as coronary dissection, occlusion of a major epicardial artery or graft, side-branch occlusion thrombus, disruption of collateral flow, or distal embolization.

### Statistical Analysis

We used *t*-test and chi-square test to compare variables between the positive cTNT group and the negative cTNT group. ANOVA analysis was used for the pairwise comparison among the three group. A time-to-event model was used to analyze the primary endpoint between the two groups. Log rank test was done to compare the significance of the Kaplan–Meier curve. All statistical analyses were performed using Stata 23.0 (Stata Corp, 2015).

## Results

**Figure 1:**
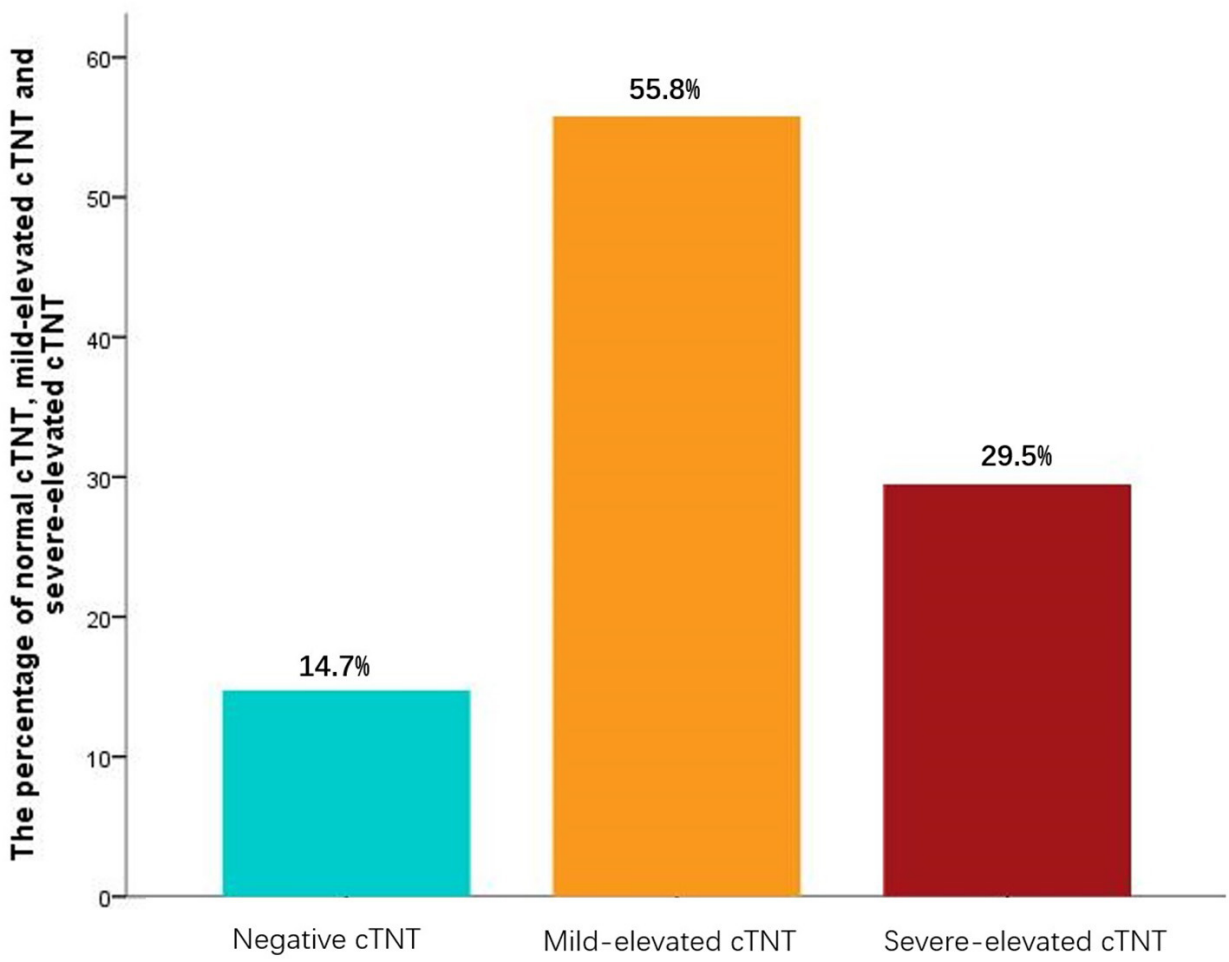
Postprocedure cardiac troponin T (cTNT). Mild-elevated cTNT is defined as post-cTNT higher than 0.03 but lower than 0.09 μg/L. Severe-elevated post-cTNT is defined as ≥0.09 μ g/L. The median and quartile range of postprocedure cTNT was 0.037 μg/L (0.064–0.108 μg/L).

A total of 190 patients were enrolled in the study. About 85.3% of the patients were in the group of elevated cTNT, while 14.7% of them were in the group of normal cTNT, among which 55.8% had a mild increase and 29.5% had a severe increase in cTNT (Figure 1). The median and quartile range of postprocedure cTNT was 0.037 μg/L (0.064–0.108 μg/L).

### Baseline Characteristics

The baseline characteristics between the normal cTNT and elevated cTNT groups are presented in Table 1. Compared with the normal cTNT group, patients in the elevated cTNT group have lower GFR levels (72.28 ± 18.84 vs. 86.79 ± 12.64, *p* = 0.017). More patients in the elevated cTNT group have hypertension (67.3 vs. 46.4%, *p* = 0.053) and history of atrial fibrillation ablation (14.5 vs. 3.6%, *p* = 0.135), although there was no significant difference.

**Table 1:** Baseline characteristics between the normal cardiac troponin T (cTNT) and elevated cTNT groups.

	**Normal cTNT**	**Elevated cTNT**	***p***
	***n* = 28**	***n* = 162**	
Age	68.90 ± 9.72	66.43 ± 7.31	0.393
Male	16 (57.1%)	95 (58.6%)	0.882
Hypertension	13 (46.4%)	109 (67.3%)	0.053
Diabetes	5 (17.9%)	34 (21.0%)	0.805
Hyperlipidemia	0	10 (6.2%)	0.363
History of cerebrovascular disease	15 (53.6%)	69 (42.6%)	0.308
Coronary heart disease	4 (14.3%)	27 (16.7%)	1.000
Structural heart disease	5 (17.9%)	29 (17.9%)	1.000
Heart failure	2 (9.5%)	19 (11.7%)	0.745
History of radiofrequency ablation	1 (3.6)	23 (14.5%)	0.135
History of malignant tumor	1 (3.6%)	4 (2.5%)	0.554
Hemoglobin (HB)	136.96 ± 17.78	135.83 ± 16.51	0.761
White blood cell (WBC)	5.37 ± 1.69	6.18 ± 1.84	0.430
Endogenous glomerular filtration rate (eGFR)	86.79 ± 12.64	72.28 ± 18.84	0.017
Left ventricular ejection fraction (LVEF)	60.0 ± 14.0	63.10 ± 9.23	0.940

Other characteristics like age, sex, history of hyperlipidemia, coronary artery disease, and heart failure are distributed evenly between the two groups.

### Intraoperative Information Between the Normal Cardiac Troponin T and the Elevated Cardiac Troponin T Group

We further analyzed the characteristics of the LAAO procedure between the two groups. The procedure length had no significant difference in the different cTNT group [60.0 (57.5–73.8) vs. 60.0 (50.0–70.0), *p* = 0.923]. It is worth noting that the longer exposure time was in the higher cTNT [11.0 (10.0–17.2) vs. 18.4 (13.6–25.5), *p* < 0.01], which was the same as the dosage of contrast [277 (194–360) vs. 406.0 (342.5–590.0), *p* < 0.01]. More patients implanted with the Watchman device had elevated cTNT than patients implanted with the Lambre device (89.2 vs. 76.7%, *p* = 0.029). Times for changing LAAO devices, morphological characteristics of the left atrium, length and depth of atrial atrium, and heart rate had no statistical difference between the two groups (Table 2).

**Table 2:** Intraoperative information between the normal cTNT and elevated cTNT groups.

	**Normal cTNT**	**Elevated cTNT**	***p***
	***N* = 28**	***N* = 162**	
Procedural length (min)	60.0 (57.5–73.8)	60.0 (50.0–70.0)	0.923
Exposure time (min)	11.1 (10.0–17.2)	18.4 (13.56–25.48)	<0.01
Dosage of contrast (mGy)	277 (194–360)	406.0 (342.5–590.0)	<0.01
Times for changing left atrial appendage occlusion (LAAO) device (≥2)	2 (7.4%)	13 (8.1%)	0.632
**Types of left atrial appendage**			0.029
Watchman	14 (10.8%)	116 (89.2%)	
Lambre	14 (23.3%)	46 (76.7%)	
**Morphological characteristics of the left atrium**			0.472
Cauliflower type	21 (14.9%)	120 (85.1%)	
Chicken wing type	1 (5.6%)	17 (94.4%)	
Vane type	1 (25.0%)	3 (75.0%)	
The cactus type	1 (50.0%)	1 (50.0%)	
Others	4 (16.0%)	21 (84.0%)	
Length of atrial atrium (mm)	21.92 ± 5.82	21.47 ± 5.13	0.894
Depth of atrial atrium (mm)	32.50 ± 7.77	31.24 ± 7.66	0.387
Heart rate (bpm)	83.70 ± 15.67	80.35 ± 13.13	0.940

### Watchman Device and Lambre Device

In the patients receiving the Watchman device, we found that shapes of LAA were associated with elevated cTNT, among which chicken wing type was more likely to have a higher cTNT level (100 vs. 0%). We found no difference in the size of Watchman, compression ratio, size of atrial atrium, and residual regurgitation between the two groups (Table 3).

**Table 3:** Information of the Watchman group.

	**Normal cTNT**	**Elevated cTNT**	***p***
	***N* = 14**	***N* = 116**	
**Intraoperative information**			
Size of Watchman (mm)	26.21 ± 7.68	28.51 ± 4.64	0.496
Compression ratio	15.66 ± 3.25	16.59 ± 4.92	0.147
Length of atrial atrium (mm)	19.31 ± 6.59	20.60 ± 4.64	0.558
Depth of atrial atrium (mm)	33.69 ± 10.37	30.94 ± 7.53	0.437
**Morphological characteristics of the left atrium**			0.045
Cauliflower type	10 (10.8%)	83 (89.2%)	
Chicken wing type	0	10 (100%)	
Vane type	0	2 (100%)	
The cactus type	1 (100%)	0	
Others	3 (12.5%)	21 (87.5%)	
Residual regurgitation	0	1 (89.0%)	0.63

For the patients receiving the Lambre device, a larger diameter of the outer disc of the device (35.04 ± 4.04 vs. 36.86 ± 1.88, *p* = 0.001) and deeper LAAs (31.31 ± 3.88 vs. 31.96 ± 8.01, *p* = 0.027) were more prevalent in the elevated cTNT group. Different shapes of the left atrium were distributed equally in the different cTNT groups (Table 4).

**Table 4:** Information of the Lambre group.

	**Normal cTNT**	**Elevated cTNT**	***p***
	***N* = 14**	***N* = 46**	
**Intraoperative information**			
Diameter of inner disc (mm)	29.39 ± 4.50	26.44 ± 6.32	0.215
Diameter of outer disc (mm)	35.04 ± 4.04	36.86 ± 1.88	0.001
Length of atrial atrium (mm)	24.54 ± 3.50	23.57 ± 5.66	0.058
Depth of atrial atrium (mm)	31.31 ± 3.88	31.96 ± 8.01	0.027
Heart rate (bpm)	77.91 ± 11.23	80.25 ± 14.30	0.428
**Morphological characteristics of the left atrium**			0.305
Cauliflower type	11 (22.9%)	37 (77.1%)	
Chicken wing type	1 (12.5%)	7 (87.5%)	
Vane type	1 (50.0%)	1 (50.0%)	
The cactus type	0	1 (100%)	
Others	1 (100%)	0	
Residual regurgitation	2 (14.3%)	4 (8.7%)	0.556

### Follow-Up Results

**Figure 2:**
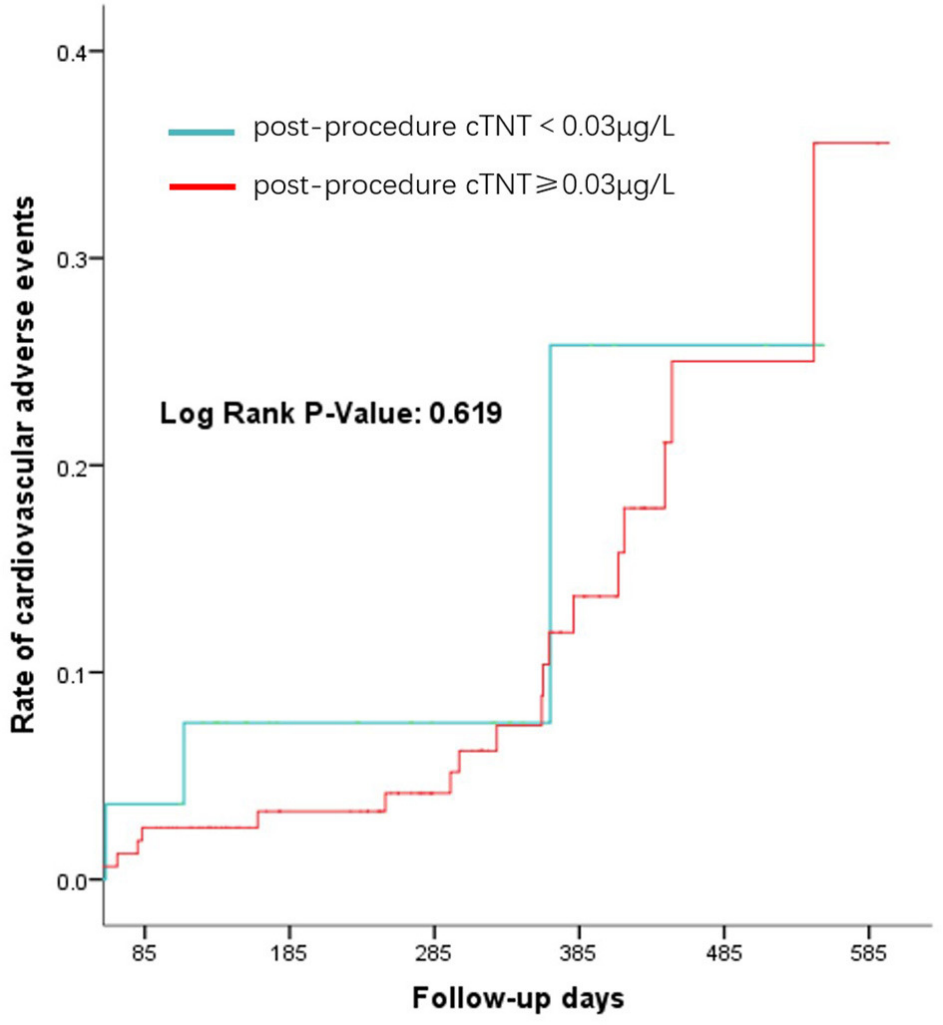
Long-term cardiovascular events after left atrial appendage occlusion (LAAO).

Regarding peri-procedural complications, there is no significant difference between the elevated cTNT group and the normal cTNT group, regardless of which device was used (Table 5). There was also no difference in the incidence of 1-year cardiovascular events between the two groups (p-value: 0.619) (Figure 2).

**Table 5:** Periprocedural complications between the positive cTNT group and the negative cTNT group.

	**Normal cTNT**	**Elevated cTNT**	***p***
**In-hospital adverse events-overall**	***N*** **=** **28**	***N*** **=** **162**	
Pericardial tamponade	1 (3.5%)	6 (6.5%)	1.000
Adverse events	1 (3.5%)	13 (8.0%)	0.697
**In-hospital adverse events-Watchman**	***N*** **=** **14**	***N*** **=** **116**	
Pericardial tamponade	0	3 (2.7%)	0.400
Adverse events	0	8 (6.9%)	0.170
**In-hospital adverse events-Lambre**	***N*** **=** **14**	***N*** **=** **46**	
Pericardial tamponade	1 (7.1%)	3 (6.5%)	0.935
Adverse events	1 (7.1%)	5 (10.9%)	0.674

## Discussion

Our study found that 85.3% (162/190) of LAAO patients had elevated cTNT after the procedure. This elevation might not be related to the complications of left atrial appendage occlusion and cardiac outcomes within 1 year of follow-up. Furthermore, eGFR at baseline, exposure time, dosage of contrast, type of LAAO device, and LAA morphology were found to be independently associated with cTNT elevation after the procedure. Our study is the first we know to explore the incidence, significance, and risk factors of elevated cTNT for patients after LAAO.

In our study, longer exposure time and higher dose of contrast were associated with higher levels of cTNT, indicating that the cTNT elevation in LAAO was probably related to the procedure itself, since patients with elevated cTNT had no evidence of chest pain, and hemodynamic or respiratory distress afterward (17). The cause of cTNT elevation in our study was likely by the implantation of a LAAO device, which was supported by the positive association between exposure time and cTNT elevation since longer exposure time often suggests more complexity of the procedure and more times of attempts to place a LAAO device, leading to more myocardial damage. However, the longer procedure time was not associated with higher cTNT levels in our study, and the reasons may be that procedure time have more confounding factors such as the puncture time, the skill of the operator, the team coordination, and so on.

What is more, the types of LAAO device affected the level of cTNT. The post-cTNT in the Watchman group was higher than that of the Lambre group. Like other intra-cardiac procedures, LAAO could cause potential cardiac damage by anchoring in the myocardium, decompression of the device, and mechanical compression of the LAA (18). For the Watchman device, certain shapes of LAA correlates with the higher level of cTNT. However, for the Lambre device, the diameter of the outer disc of the device and the length of the left atrial appendage are associated with higher levels of cTNT. The reasons for those results were listed as follows. First, designs of the device could affect postprocedure cTNT levels. For example, the design of the transport system, release mechanism, and the way of the device binding to the left atrial appendage may explain the level of post-cTNT after LAAO. The Watchman has a barb, which is fixed in the left atrial appendage by relying on the barb, which will inevitably cause myocardial injury. In addition, it needs to be pulled before releasing the occlusion, and if the position is not proper, it will need to be adjusted repeatedly, which may increase the injury of the operation; The Lambre occluder is a disc occluder, which will not directly damage the myocardium in most cases, but repeated extrusion or friction may cause damage (18). Second, morphology of LAAs could affect postprocedure cTNT level. The shape of LAA mainly affects postprocedure cTNT in patients receiving the Watchman device. With regard to the Lambre device group, patients who had larger disc diameter and deeper LAA are more likely to have positive postprocedure cTNT. It is likely because the Watchman device requires more for the left atrial morphology, while the Lambre device does not (18–21). The Watchman device becomes spherical after expansion, and when the atrial shape does not match the shape of the Watchman device, myocardial injury is likely to occur, which has been mentioned before. On the contrary, the Lambre device is highly adaptable to different LAA morphologies because of its disc occluder. It can be quite useful in patients with difficulty anatomies (21). The Lambre device is embedded in the atrial wall by hook and parachute, so the requirement of atrial morphology is not as high as that of the Watchman, but the outer disk size and atrial diameter of the Lambre device are accountable for the degree of myocardial injury.

In our study, patients with lower eGFR were also found to be related to higher incidence of cTNT elevation after the procedure. Renal dysfunction is one of the major reasons for cTNT elevation except for acute myocardial infarction (22). cTNT elevation is common among patients with renal failure, indicating that cTNT was cleared by the kidneys (23). In addition, cTNT elevations in these patients could be due to increased cTNT release linked to cardiac stress, called the cardiorenal syndrome (24). Martens et al. found that eGFR was independently associated with cTNT levels, even when eGFR levels do not fulfill the CKD criteria (25). Our results were consistent with a previous study that eGFR was associated with cTNT elevation after LAAO in the condition that the means of eGFR of the two groups do not meet the eGFR criteria.

cTNT elevation has been associated with poor prognosis in many conditions (26, 27). However, the mild and transient elevation of cTNT in our study did not contribute to the poor prognosis, indicating that the increase in cTNT is mostly transient myocardial injury caused by occluder implantation, which does not affect cardiac ejection function and ventricular survival area. Our results were consistent with previous studies (28, 29). Similarly, all patients in our study were followed up for 1 year, and the cTNT elevation had no relation to the complications of the procedure or cardiac outcomes. Thus, it is demonstrated that cTNT elevation after LAAO was not associated with adverse clinical events and complications within 1 year follow-up in this relatively large cohort.

## Limitations

The limitations of our analyses are listed as follows: First, the sample of this study is a little small, which decreases the reliability level of the conclusion. Second, our study is a single-center study, which may result in selection bias. Third, we were unable to rule out the influence of differing skill levels of different operators involved in the procedure on cTNT elevation.

## Conclusion

LAAO was found to be commonly accompanied with cTNT elevation, which might not to be related to the complications and adverse cardiac outcomes within 1 year of follow-up. Moreover, eGFR at baseline, exposure time, dosage of contrast, types of LAAO device, and LAA morphology could contribute to cTNT elevation.

## Data Availability Statement

The raw data supporting the conclusions of this article will be made available by the authors, without undue reservation.

## Ethics Statement

The studies involving human participants were reviewed and approved by Institutional Review Board of Zhongshan Hospital, Fudan University. The patients/participants provided their written informed consent to participate in this study. Written informed consent was obtained from the individual(s) for the publication of any potentially identifiable images or data included in this article.

## Author Contributions

XiaW, XC, YY, DZ, and QC conceptualized and designed the study. QC and DZ provided administrative support. XiaW and QC analyzed and interpreted the data. XiaW, YY, and QC wrote the manuscript. All authors gave the final approval of the manuscript and provided the study materials or patients and collected and assembled the data.

## Funding

This study was supported by the National Natural Science Foundation of China (82000267, 81870197, 81500191, 81900260, and 8180020174) and The Health Science and Technology Project of Shanghai Pudong New Area Health Commission (PW2019A-13).

## Conflict of Interest

The authors declare that the research was conducted in the absence of any commercial or financial relationships that could be construed as a potential conflict of interest.

## Publisher's Note

All claims expressed in this article are solely those of the authors and do not necessarily represent those of their affiliated organizations, or those of the publisher, the editors and the reviewers. Any product that may be evaluated in this article, or claim that may be made by its manufacturer, is not guaranteed or endorsed by the publisher.

## References

[1] Thygesen K Alpert JS Jaffe AS Chaitman BR Bax JJ Morrow DA. Fourth universal definition of myocardial infarction. J Am Coll Cardiol. (2018) 72:2231–64. 10.1161/CIR.0000000000000617 30153967

[2] Zeitouni M Silvain J Guedeney P Kerneis M Yan Y Overtchouk P. Periprocedural myocardial infarction and injury in elective coronary stenting. Eur Heart J. (2018) 39:1100–9. 10.1093/eurheartj/ehx799 29365133

[3] Harada Y Koskinas KC Ndrepepa G Raber L Braun S Zanchin T. Postprocedural high-sensitivity troponin T and prognosis in patients with non-ST-segment elevation myocardial infarction treated with early percutaneous coronary intervention. Cardiovasc Revasc Med. (2018) 19:480–6. 10.1016/j.carrev.2017.11.010 29292015

[4] Gahl B Gober V Odutayo A Tevaearai Stahel HT da Costa BR Jakob SM. Prognostic value of early postoperative troponin T in patients undergoing coronary artery bypass grafting. J Am Heart Assoc. (2018) 7:e007743. 10.1161/JAHA.117.007743 29487111 PMC5866325

[5] Machado MN Rodrigues FB Grigolo IH Sabbag ATR Silva OLJ Maia LN. Early prognostic value of high-sensitivity troponin t after coronary artery bypass grafting. Thorac Cardiovasc Surg. (2019) 67:467–74. 10.1055/s-0038-1675640 30485894

[6] Reichlin T Lockwood SJ Conrad MJ Nof E Michaud GF John RM. Early release of high-sensitive cardiac troponin during complex catheter ablation for ventricular tachycardia and atrial fibrillation. J Interv Card Electrophysiol. (2016) 47:69–74. 10.1007/s10840-016-0125-6 26971332

[7] Kozinski M Krintus M Kubica J Sypniewska G. High-sensitivity cardiac troponin assays: from improved analytical performance to enhanced risk stratification. Crit Rev Clin Lab Sci. (2017) 54:143–72. 10.1080/10408363.2017.1285268 28457177

[8] Yoshida K Yui Y Kimata A Koda N Kato J Baba M. Troponin elevation after radiofrequency catheter ablation of atrial fibrillation: relevance to AF substrate, procedural outcomes, and reverse structural remodeling. Heart Rhythm. (2014) 11:1336–42. 10.1016/j.hrthm.2014.04.015 24732367

[9] Vasatova M Pudil R Tichy M Buchler T Horacek JM Haman L. High-sensitivity troponin T as a marker of myocardial injury after radiofrequency catheter ablation. Ann Clin Biochem. (2011) 48:38–40. 10.1258/acb.2010.009280 21098548

[10] Furniss G Shi B Jimenez A Harding SA Larsen PD. Cardiac troponin levels following implantable cardioverter defibrillation implantation and testing. Europace. (2015) 17:262–6. 10.1093/europace/euu306 25414480

[11] Brewster J Sexton T Dhaliwal G Charnigo R Morales G Parrott K. Acute effects of implantable cardioverter-defibrillator shocks on biomarkers of myocardial injury, apoptosis, heart failure, and systemic inflammation. Pacing Clin Electrophysiol. (2017) 40:344–52. 10.1111/pace.13037 28156007 PMC5395321

[12] Welsh P Preiss D Hayward C Shah ASV McAllister D Briggs A. Cardiac troponin t and troponin i in the general population. Circulation. (2019) 139:2754–64. 10.1161/CIRCULATIONAHA.118.038529 31014085 PMC6571179

[13] Willeit P Evans JDW Tschiderer L Boachie C Jukema JW Ford I. High-sensitivity cardiac troponin concentration and risk of first-ever cardiovascular outcomes in 154,052 participants. J Am Coll Cardiol. (2017) 70:558–68. 10.1016/j.jacc.2017.05.062 28750699 PMC5527070

[14] Holmes DR Turi ZG Doshi SK Sievert H Buchbinder M Mullin CM. Percutaneous closure of the left atrial appendage versus warfarin therapy for prevention of stroke in patients with atrial fibrillation: a randomised non-inferiority trial. Lancet. (2009) 374:534–42. 10.1016/S0140-6736(09)61343-X 19683639

[15] Reddy VY Doshi SK Sievert H Buchbinder M Neuzil P Huber K. Percutaneous left atrial appendage closure for stroke prophylaxis in patients with atrial fibrillation: 2.3-year follow-up of the PROTECT AF (Watchman left atrial appendage system for embolic protection in patients with atrial fibrillation) trial. Circulation. (2013) 127:720–9. 10.1161/CIRCULATIONAHA.112.114389 23325525

[16] Yves-Laurent Bayard HO Neuzil P Thuesen L Pichler L Rowland E Ramondo A. PLAATO (Percutaneous Left Atrial Appendage Transcatheter Occlusion) for prevention of cardioembolic stroke in non-anticoagulation eligible atrial fibrillation patients: results from the European PLAATO study. EuroIntervention. (2010) 6:220–6. 10.4244/EIJV6I2A35 20562072

[17] Franck Maziere SB Medimagh S Arthaud M Bennaceur M Riou B Ray P. Comparison of troponin I and N-terminal-pro B-type natriuretic peptide for risk stratification in patients with pulmonary embolism. Eur J Emerg Med. (2007) 14:207–11. 10.1097/MEJ.0b013e3280bef891 17620911

[18] Chen S Chun KRJ Bordignon S Weise FK Nagase T Perrotta L. Left atrial appendage occlusion using LAmbre Amulet and Watchman in atrial fibrillation. J Cardiol. (2019) 73:299–306. 10.1016/j.jjcc.2018.10.010 30583991

[19] Chen S Schmidt B Bordignon S Bologna F Nagase T Tsianakas N. Feasibility of percutaneous left atrial appendage closure using a novel LAmbre occluder in patients with atrial fibrillation: Initial results from a prospective cohort registry study. J Cardiovasc Electrophysiol. (2018) 29:291–7. 10.1111/jce.13385 29149516

[20] Fountain RB Holmes DR Chandrasekaran K Packer D Asirvatham S Van Tassel R. The PROTECT AF (WATCHMAN left atrial appendage system for embolic PROTECTion in patients with atrial fibrillation) trial. Am Heart J. (2006) 151:956–61. 10.1016/j.ahj.2006.02.005 16644311

[21] Lam YY. A new left atrial appendage occluder (Lifetech LAmbre Device) for stroke prevention in atrial fibrillation. Cardiovasc Revasc Med. (2013) 14:134–6. 10.1016/j.carrev.2013.04.003 23773494

[22] Roffi M Patrono C Collet JP Mueller C Valgimigli M Andreotti F. 2015 ESC Guidelines for the management of acute coronary syndromes in patients presenting without persistent ST-segment elevation: task force for the management of acute coronary syndromes in patients presenting without persistent ST-segment elevation of the European Society of Cardiology (ESC). Eur Heart J. (2016) 37:267–315. 10.1093/eurheartj/ehv320 26320110

[23] deFilippi C Kelley W Duh SH Hise M Christenson RH. Interpreting cardiac troponin results from high-sensitivity assays in chronic kidney disease without acute coronary syndrome. Clin Chem. (2012) 58:1342–51. 10.1373/clinchem.2012.185322 22791885

[24] Palazzuoli A Ronco C Maisel A. Clinical relevance of biomarkers in heart failure and cardiorenal syndrome: the role of natriuretic peptides and troponin. Heart Fail Rev. (2014) 19:267–84. 10.1007/s10741-013-9391-x 23563622

[25] Martens RJ Honney K Kooman JP Stehouwer CD Tan FE BekersO. Estimated glomerular filtration rate and albuminuria are associated with biomarkers of cardiac injury in a population based cohort study: the Maastricht Study. Clin Chem. (2017) 63:887–97. 10.1373/clinchem.2016.266031 28213568

[26] Mannu GS Honney K Spooner R Clark AB Bettencourt-Silva JH Zaman MJ. Incidentally raised cardiac troponin I has a worse prognosis in older patients compared to those with normal cardiac troponin I and patients with acute coronary syndrome: a cohort study. Gerontology. (2016) 62:581–7. 10.1159/000444083 27007948

[27] Thielmann M Massoudy P Neuhauser M Knipp S Kamler M Piotrowski J. Prognostic value of preoperative cardiac troponin I in patients with non-ST-segment elevation acute coronary syndromes undergoing coronary artery bypass surgery. Chest. (2005) 128:3526–36. 10.1016/S0012-3692(15)52926-7 16304309

[28] Boersma LV Schmidt B Betts TR Sievert H Tamburino C Teiger E. Implant success and safety of left atrial appendage closure with the WATCHMAN device: peri-procedural outcomes from the EWOLUTION registry. Eur Heart J. (2016) 37:2465–74. 10.1093/eurheartj/ehv730 26822918 PMC4996118

[29] Chen X Yu Z Bai J Hu S Wang W Qin S. Troponin T elevation after permanent pacemaker implantation. J Interv Card Electrophysiol. (2017) 49:211–8. 10.1007/s10840-017-0247-5 28417288

